# Psychological Well-Being, Self-Esteem, and Body Image Satisfaction Among Pregnant Tunisian Women: A Study from Monastir Maternity Department

**DOI:** 10.1192/j.eurpsy.2025.1291

**Published:** 2025-08-26

**Authors:** A. Hadj Salah, D. Bouhlel, I. Batbout, A. Haouala, M. Ben Mbarek, F. Zaafrane, A. Mhalla, B. Amamou

**Affiliations:** 1 psychiatry; 2 gynecology, Fattouma Bourguiba Hospital, Monastir, Tunisia

## Abstract

**Introduction:**

Pregnancy induces significant physiological and psychological changes. This study evaluates self-esteem, body image satisfaction, and psychological well-being among pregnant women at Monastir Maternity Center, Tunisia.

**Objectives:**

1. To assess self-esteem and body image satisfaction among pregnant women.
2. To evaluate the psychological well-being and the adequacy of support provided during pregnancy.

**Methods:**

Conducted over two months (March-April 2022) with 62 participants (88.57% response rate) at Monastir Maternity Center. Self-esteem was measured using the Rosenberg Self-Esteem Scale (RSES), and body image satisfaction was assessed with the Body Image States Scale (BISS). Sociodemographic, obstetric, and psychosocial variables were collected.

**Results:**

The study included 62 participants, with an average age of 31.68 ± 7.49 years. Most participants (62.9%) were stay-at-home individuals, and two-thirds reported their current pregnancy as desired.

Body image satisfaction, measured by the Body Image States Scale, averaged 5.32 ± 3.67, indicating moderate satisfaction.

Self-esteem was low in 79% of participants, while 74.2% had no depressive symptoms and 90.3% had no anxiety symptoms.

Half of the participants (50%) reported their psychological well-being was addressed during pregnancy, with the majority (41.9%) receiving support from family members. Three-quarters (75%) considered pregnancy consultations the best time to discuss psychological issues. Almost all (98.4%) wanted additional psychological support. Over half (56.5%) talked about their pregnancy difficulties with someone, while 22.6% did not address them, and 21.0% did not experience any difficulties.

**Conclusions:**

The study highlights a significant prevalence of low self-esteem and moderate body image satisfaction among pregnant women.. The study also reveals that while many women discuss their difficulties with others, there remains a gap in addressing psychological issues adequately, particularly considering the majority of participants desire more support.

**Disclosure of Interest:**

None Declared

